# A cross-sectional analysis of zinc and copper levels and their relationship to cardiovascular disease risk markers in Qatar biobank participants

**DOI:** 10.3389/fcvm.2023.1305588

**Published:** 2024-01-05

**Authors:** Abdelhamid Kerkadi, Hicham Raïq, Mohammad Shoaib Prince, Loulia Bader, Abderrezzaq Soltani, Abdelali Agouni

**Affiliations:** ^1^ Department of Patient Care & Health Technology, College of Health Sciences, University of Doha for Science and Technology, Doha, Qatar; ^2^ Department of Social Sciences, College of Arts and Sciences, Qatar University, Doha, Qatar; ^3^ Sport and Wellness Department, University of Doha for Science and Technology (UDST), Doha, Qatar; ^4^ Department of Pharmaceutical Sciences, College of Pharmacy, QU Health, Qatar University, Doha, Qatar; ^5^ College of Pharmacy, QU Health, Qatar University, Doha, Qatar; ^6^ Office of Vice President for Health & Medical Sciences, Qatar University, Doha, Qatar; ^7^ Office of Vice President for Research & Graduate Studies, Qatar University, Doha, Qatar

**Keywords:** zinc, copper, zinc/copper ratio, cardiovascular disease risk, trace elements

## Abstract

Cardiovascular diseases (CVD) are the leading cause of mortality and morbidity worldwide. Dietary intake, particularly zinc (Zn) and copper (Cu) has been strongly associated with CVD. These trace elements play a crucial role in human enzyme activity, suppressing inflammation, catalyzing lipid metabolism enzymes, reducing oxidative stress, and regulating glucose metabolism. However, imbalances in these elements are linked to cardiovascular disturbances. Thus, this study aimed to investigate the association between circulating levels of Zn, Cu, and Zn/Cu ratio with CVD risk factors in the Qatari population. Bivariate logistic regression, adjusted for age, nationality, gender, and education was performed to examine the impact of Zn, Cu, and Zn/Cu ratio (as independent variables) on major CVD risk markers (as dependent variables). Participants in the highest Zn tertiles (T2 and T3) were at greater odds ratio (OR) of unfavorable metabolic functions such as elevated HbA1C [OR = 2.5, *p* = 0.015 (T2) and OR = 3.2, *p* = 0.002 (T3)], triglycerides [OR = 2.17, *p* = 0.015 (T2), and TyG index [OR = 2.21, *p* = 0.004 (T2), and OR = 2.67, p < 0.001 (T3)] compared to T1. Conversely, they had significantly lower ORs for prolonged prothrombin time [OR = 0.37, *p* = 0.001 (T3)]. Higher levels of Cu (T2 and T3) had higher OR for elevated HDL-C levels [OR = 1.69, *p* = 0.046 (T2), and OR = 2.27, *p* = 0.002 (T3)] and lower OR for elevated levels of triglycerides (OR = 0.4, *p* = 0.009, T3), diastolic blood pressure [OR = 0.41, *p* = 0.024 (T2), and OR = 0.47, *p* = 0.049 (T3)], and creatinine kinase (OR = 0.27, *p* = 0.014, T3) compared to T1. Higher levels of Cu (T2 and T3) were associated with a higher risk for elevated fibrinogen levels [OR = 3.1, *p* = 0.035 (T2), and OR = 5.04, *p* = 0.002 (T3)]. Additionally, higher Zn/Cu ratio (T2 and T3) were associated with lower ORs for elevated fibrinogen levels [OR = 0.3, *p* = 0.005 (T2), and OR = 0.27, *p* = 0.005 (T3)] compared to T1, indicating a lower risk of developing CVD. The study reveals a link between Zn, Cu, and the Zn/Cu ratio and cardiovascular disease risk. A higher Zn/Cu ratio may protect against CVD, while elevated Cu levels are linked to obesity, fibrinogen levels, and HbA1C. Maintaining optimal levels of these trace elements, either through diet or supplementation, may help reduce CVD risk.

## Introduction

Globally, the leading cause of mortality and morbidity is cardiovascular diseases (CVD) (1, 2), accounting for 32% of all deaths in 2019, with 85% of these deaths caused by heart attacks and stroke (2). In Qatar, approximately 24% of total mortality is attributed to CVD (3–5). In 2019, the age-adjusted prevalence of coronary heart disease in Qatar was reported as 16 per 1,000 population (6). While the CVDs are more prevalent in low- and middle-income countries, with over three-quarters of CVD deaths occurring in these regions (3). CVDs also contribute significantly to premature deaths, with 38% of non-communicable disease-related premature deaths in 2019 attributed to CVDs (2–4). In the Qatar Health Strategy (2017–2022), it was reported that deaths from CVD in 2011–2013 were 8.3 per 100,000 Qatari males and 8.3 per 100,000 non-Qatari males. However, those numbers increased in those above the age of 45 years to be 247 per 100,000 Qatari males. Moreover, in 2014 14.4% of overall years of life lost (YLL) in Qatar were attributed to cardiovascular and circulatory disease (7). According to a recent report from the American Heart Association (AHA) (2023), CVD is a leading cause of mortality worldwide, with an expected 19.05 million deaths in 2020, an increase of 18.71% since 2010 (2). Globally, the crude prevalence of CVD cases increased by 29.01% between 2010 and 2020 (2). The risk of cardiovascular events is increased by the presence of cardiometabolic risk factors such as hypertension (4), dyslipidemia (8), central obesity (9), diabetes (10), and inflammation (4). In the Middle East, CVDs account for 34% of all deaths. The region's CVD prevalence was reported to be 13.7%, with CVD causing 24% of all deaths in 2016 (3, 5). Behavioral risk factors such as tobacco use, unhealthy diet and obesity, physical inactivity, and harmful alcohol use are modifiable risk factors for CVD (1, 11). Among several established risk factors, food habits or dietary intake have been strongly associated with CVD (11–13). The circulating levels of trace elements such as zinc (Zn) (14–16), copper (Cu) (17–20), and the zinc-to-copper (Zn/Cu) ratio (21, 22) have also been associated with CVD.

The human body requires trace elements such as Zn and Cu to function (23). Zn plays a crucial role in the functioning of multiple human enzymes and factors (24); it is essential for over 300 enzymes and 2,000 transcription factors in the human body (25). Zn plays a key role in suppressing inflammation (14, 26), catalyzing lipid metabolism enzymes, and reducing oxidative stress (13, 15, 16, 27). Moreover, muscle and fat cells utilize Zn as a cofactor for various intracellular enzymes to facilitate glucose metabolism (28). Additionally, Zn regulates the production of insulin receptors and their signal transduction mechanism (29). Zn Deficiencies have been linked to a variety of congenital cardiac abnormalities, making it a critical nutrient for normal heart development throughout embryonic and fetal stages. Zn is necessary for monocyte-related processes in atherosclerosis, such as endothelium activation, transformation, adhesion, into macrophages, and development of foam cells through the ingestion of oxidized low-density lipoprotein (LDL) particles (25). Intracellular Zn levels have been associated with heart failure and arterial hypertension by elevating intracellular calcium levels through several mechanisms and acting identically to calcium as a second messenger (30). Conversely, extracellular Zn has the opposite impact by inhibiting calcium channels, which may explain why individuals with hypertension have lower blood zinc levels but higher in cardiomyocytes and erythrocytes. Moreover, Individuals with ischemia/myocardial infarction, conduction abnormalities, congestive heart failure, and heart transplants had lower blood Zn levels. In animal experimental models, the administration of Zn during reperfusion improved myocardial recovery by up to 100% and was shown to be twice as likely to reduce arrhythmias (31).

Cu is an essential micronutrient for the human body as it is crucial for the functioning of enzymes that facilitate oxidation-reduction reactions (18, 32). It is a cofactor for several antioxidant (18) and oxidoreductive enzymes (17) that lower the risk of cardiovascular disease, enhance cardiovascular function, and control fat metabolism (33, 34). Deficiency in Cu may lead to oxidative damage because it reduces the activity of copper-dependent antioxidant enzymes such as Cu/Zn superoxide dismutase (SOD), ceruloplasmin, and cytochrome c oxidase (33). These enzymes are essential for eliminating reactive oxygen species (ROS) (35). Cu is essential for reducing the activity of the angiotensin-converting enzyme activity (ACE) (36); it also helps in maintaining healthy blood vessels and controlling blood pressure. Animal studies have indicated that a diet lacking in Cu may cause heart disease, high blood pressure, and elevated cholesterol and triglyceride levels (37, 38). High levels of circulating Cu have been associated with the development of CVD. Nevertheless, the causal relationship between Cu and the development of atherosclerosis remains uncertain, as it is yet to be determined whether Cu directly influences atherosclerosis formation or only acts as an indicator of inflammation associated with atherosclerosis (32). The aberrant aggregation of lipoylated proteins occurs when copper ions are present, resulting in a decrease in the production of proteotoxic stress, Fe-S cluster proteins, and ultimately leading to cell death (39). Cu can induce cellular apoptosis through various mechanisms, including the generation of reactive oxygen species (ROS), activation of endoplasmic reticulum (ER) stress, and initiation of inflammatory responses (40). Cu-induced Cell death is linked to oxidative stress and inflammation, playing a significant role in the pathogenesis of cardiovascular conditions including stroke, coronary artery disease (CAD), heart failure, atherosclerosis, and ischemia-reperfusion injury (39–41).

A multitude of studies have investigated the correlation between Zn, Cu, and risk factors for CVD, encompassing lipid profiles, diabetic measures, and hypertension (3). Previously, we discussed inconsistent findings in the literature. For example, although some research has associated Zn deficiency with increased total cholesterol (TC) and LDL cholesterol (LDL-C) levels (42), other researchers reported no significant association between Zn levels and lipid profile (43), fatty acids (44) and CVD risk (45, 46). Contrarily, plasma Cu levels have been positively associated with elevated levels of TC, LDL-C, and high-density lipoproteins cholesterol (HDL-C) (47). Cu levels of more than 130 g/dl may raise the risk of hypertension by 1.99 (48). However, no connection has been identified between Cu and Zn consumption or the Cu/Zn ratio and hypertension (49, 50).

The Cu/Zn ratio, which indicates the interaction between these two trace elements, is a stronger predictor of many pathologies than individual Zn and Cu levels (51). CVD-related mortality has been associated with a high circulating Cu/Zn ratio (52, 53). Several inflammatory indicators and serum albumin concentrations were shown to correlate with the circulating Cu/Zn ratio. Moreover, this ratio was an accurate prognostic of mortality in those over 70 years of age for a period of 3.5 years (22). The plasma Cu/Zn ratio was shown to be higher in individuals with stable CVD compared to those without, mostly owing to increased circulating Cu levels. However, a progressive decline in plasma Zn levels was suspected to be the primary driver for the Cu/Zn variations seen with advancing age. The Cu/Zn ratio is an important clinical biomarker and a predictor of all-cause death in those over 70 years old. Qatar has seen a rise in obesity and non-communicable diseases due to dietary and lifestyle changes (3). Therefore, it is essential to understand the causes associated with the increase in cardiovascular risk. To the best of our knowledge, no research has been conducted in Qatar to investigate the relationship between trace mineral levels (Zn and Cu) and the risk of CVD in the adult population.

The current study was designed to evaluate the association between Zn, Cu, Zn/Cu ratio, and CVD risk markers in a well-characterized large sample size of participants from Qatar Biobank. We hypothesize that disturbed circulating levels of Zn and Cu are associated with cardiovascular disease risk markers. Previously, we demonstrated that elevated levels of Cu in the bloodstream have a protective effect on diastolic blood pressure (DBP), high- HDL-C, and metabolic syndrome (MetS) (3). Conversely, a high ratio of Zn/Cu in the bloodstream is linked to an increased risk of having low HDL-C and MetS.

## Methods

### Study population

This is a retrospective observational study investigating the association between the mineral status of Zn, Cu, their ratio, and key cardiometabolic markers in the Qatari population. Qatari nationals and long-term residents, who have lived in the country for at least 15 years, were recruited from Qatar Biobank (QBB). QBB is a national platform that collects biospecimen, clinical and biochemical data, and health and lifestyle information from a substantial segment of the population of Qatar. This study included adult men and women, aged 18 or older, with detailed data on mineral status (Zn and Cu) in addition to metabolic and CVD risk markers as described below. Patients were excluded from the study if they had been diagnosed with a non-communicable chronic disease (e.g., cancer, diabetes, or CVD), were taking mineral supplements or prescription drugs, had pacemakers, were pregnant, or were breastfeeding. The management team of QBB conducted a comprehensive search in the master database, resulting in the identification of 575 individuals with documented levels of Cu and Zn. A total of 138 samples were excluded from the study due to their failure to meet the predetermined inclusion criteria. In this study, a total of 437 adult volunteers were included. All study participants have previously provided written informed consent to volunteer for the QBB program. The research investigation received approval from the QBB-IRB under the reference number Ex-2020-RES-ACC-0215-0125.

The research investigation centered on a range of metabolic and cardiovascular indicators, encompassing body mass index (BMI), waist circumference (WC), fasting serum glucose, insulin, HbA1c, C-peptide, lipid profile (triglycerides, total cholesterol, low-density lipoprotein cholesterol, and high-density lipoprotein cholesterol), systolic blood pressure (SBP), diastolic blood pressure (DBP), and pulse rate (PR). Furthermore, the study incorporated findings from coagulation assessments, including prothrombin time (PT), activated partial thromboplastin time (aPTT), and international normalization ratio (INR). The investigation also involved the examination of cardiac indicators, including myoglobin blood levels, N-terminal (NT)-pro hormone B-type natriuretic peptide (NT-proBNP), and Creatinine Kinase. The concentrations of Zn, Cu, and the ratio of Zn to Cu (Zn/Cu) were also evaluated in connection to the structure and function of the common carotid arteries (both left and right) using three-dimensional Carotid Doppler ultrasonography. The Carotid Doppler ultrasonography technique is employed to assess various characteristics that indicate the level of resistance to blood flow, as well as the stiffness or constriction of the carotid artery. The criteria adopted in this study included the pulsatility index (PI), resistivity index (RI), systolic to diastolic velocity ratio (SDVR), and intima/media thickness (IMT). Elevated values of PI, RI, or SDVR may be indicative of heightened arterial stiffness or constriction, whilst a raised IMT may be suggestive of an augmented susceptibility to CVD. These indicators possess the potential to yield valuable insights in the evaluation of carotid artery disease and have the capacity to aid in the identification of individuals who are at an elevated risk of experiencing cardiovascular events (54–56).

Additionally, the investigation also examined the correlation between levels of Zn, Cu, and the ratio of Zn to Cu (Zn/Cu) and indications of arterial stiffness. The study participants’ pulse power index (PPI) and pulse wave velocity (PWV) were assessed using a viacorder (Viacor CardioMPO), which is a device utilized to capture arterial pulse waveforms at two distinct locations on the body. The calculation of PPI involves multiplying the heart rate by the stroke volume, whereas PWV represents the velocity at which the arterial pulse wave propagates between two specified locations. Both metrics play a crucial role in assessing arterial stiffness and can aid in the identification of individuals who are at an elevated risk of developing cardiovascular disease.

### Anthropometric and biochemical measurements

At the QBB clinic, data collection was conducted by technicians and nurses who possessed the necessary qualifications and received appropriate training. Measurements of height and weight were obtained using a calibrated scale and a wall-mounted stadiometer (Seca, Hamburg, Germany). The individuals were instructed to wear minimal clothing and remove their footwear during the measurements. Non-stretchable tape (Seca, Hamburg, Germany) was used to measure the waist circumference above the iliac crest at the level of the umbilicus. Systolic and diastolic blood pressure readings were averaged from three separate readings using a mercury sphygmomanometer. A digital approach was used to determine the pulse rate. After an overnight fast, blood samples were taken from each participant. Analyses of clinical samples were done at the clinical chemistry labs of Hamad Medical Corporation (HMC). Standard automated laboratory protocols were applied to assess fasting glucose, hemoglobin A1c, insulin, TC, HDL-C, and triglycerides (TG) using Hitachi-917 analyzers (Gmbh Diagnostic, Mannheim, Germany). LDL-C was determined using the Friedewald formula (57). Insulin and C-peptide concentrations and were measured with enzyme-linked immunosorbent assay (ELISA) kits (Mercodia, Uppsala, Sweden). The study assessed several coagulation tests, PT, aPTT, and INR, which were measured using standard laboratory techniques. Cardiac markers including serum levels of myoglobin, NT-proBNP, and creatinine kinase were also measured using standard laboratory assays.

The following markers and ratios were determined using the following formulas:

• Triglyceride to glucose index (TyG): Ln [TG (mg/dl) × glucose (mg/dl)/^2^]. TyG is used as a marker of insulin resistance and metabolic syndrome (58, 59).
• HOMA-IR score: fasting glucose (mg/dl) × fasting insulin (U/ml)/405. HOMA-IR score indicates insulin resistance (60).
• TG/HDL-C ratio: TG (mg/dl)/HDL-C (mg/dl). TG/HDL-C ratio is used as a marker of atherogenic dyslipidemia (61).
• The 10-year atherosclerotic cardiovascular disease (ASCVD) risk score: is used to estimate an individual’s risk of ASCVD within the next 10 years. The risk score was calculated only for individuals 40–79 years old as follows: Risk Score = −29.61 + (age × 1.15) + (if male, 4.48; if black, 4.28) + (TC × 0.18)—(HDL-C × 0.45) + (systolic blood pressure × 0.13) + (if on blood pressure medication, 1.96) + (if diabetic, 8.94) + (if current smoker, 7.67) (62).

### Zn and Cu measurements in plasma

Analysis was conducted at the clinical chemistry lab facilities of Hamad Medical Corporation (HMC). Total Cu and Zn were determined using the Inductively Coupled Plasma Mass Spectrometry (ICP-MS) method with external calibration. A diluent comprising Triton X-100 (0.05%), ammonium hydroxide (NH4OH) (1%), ethylenediaminetetraacetic acid (EDTA; 0.05%), and butanol (2%) was used to dilute plasma samples (50 L) to 1:100. (Sigma–Aldrich, Taufkirchen, Germany). Due to potential differences in ionization efficiency resulting from differences in carbon content between samples and standards (63, 64), butanol was included in the analysis. To correct the instrument drift, rhodium (Rh) was utilized as an internal standard for precision. Using 10,000 g/ml Cu (catalog number ICP-129; Agilent) and Zn (catalog number ICP-130; Agilent) calibration standards, an analytical measurement range of 0–5,000 ng/ml was established. Both samples and standards were analyzed using a ThermoScientific iCAP Q ICP-MS (ThermoScientific, Waltham, MA, USA). Seronorm trace elements serum levels 1 (cat. no. 201405) and 2 (cat. no. 203105) (Sero AS, Norway) were used as recognized standard reference material for quality control and to assure the accuracy of methods. These materials are human serum accuracy control standards that are used in the analysis of trace elements. Every calibration and sample analysis batch included examining reconstituted lyophilized reference material prepared per manufacturer specifications.

### Statistical analysis

All analyses were conducted with Stata/MP 17.0 software. *P* values of ≤ 0.05 were considered significant. The analyses were conducted in four steps:

• To characterize the sample population’s demographics, descriptive statistics were used for continuous data (mean and standard deviation) and categorical data (Frequency and percentage). For each cardiovascular marker, we calculated the mean ± SD and the percentage of participants in the normal range compared to other ranges.
• A one-way ANOVA test was used to evaluate the association between the cardiovascular markers (as continuous variables) and Zn, Cu, and the Zn/Cu tertiles. The non-parametric Kruskal–Wallis test was used when the assumptions of one-way ANOVA were not met (65).
• All CVD markers were recoded into dichotomous variables (high risk and normal). High risk cut-off values for each CVD marker were set as per the following: BMI (overweight and obese are high risk), Hip/waist size (highest quintile is the high risk), fasting glucose (> 5.5 mmol/L), insulin (> 23 mIU/L), HbA1c (> 6.5%), C-peptide (> 5.19 ng/ml), total cholesterol (> 5.2 mmol/L), HDL-cholesterol (< 1.55 mmol/L), LDL-cholesterol (> 2.59 mmol/L), triglycerides (> 1.69 mmol/L), systolic blood pressure (≥ 130 mmHg), diastolic blood pressure (≥ 80 mmHg), heart rate (> 100 beats/min), Prothrombin Time (PT) (>12.5 s), international normalized ratio (INR) (> 1.1), Activated Partial Thromboplastin Time (aPTT) (>36.5 s), Fibrinogen (>4.1 g/L), Myoglobin (for male: >78 ng/ml, female > 47 ng/ml), NT-proBNP (>125 pg/ml [<75 years old], >450 pg/ml [≥75 years old]), and Creatine Kinase (Male: >232 U/L, Female: >160 U/L), HOMA-IR: (≥ 2.28), TyG index (≥ 8.65), TG/HDL ratio (> 6) and 10-year ASCVD risk score (> 7.5).
• Then, the relationship between each cardiovascular marker (dependent variable) and Zn, Cu, and Zn/Cu ratio [independent variable] were analyzed in a series of reduced multinomial logistic regression models adjusted for gender, nationality, age, and education. The independent variables were tested at three different levels: tertile 1 (T1), teritile 2 (T2), and tertile 3 (T3), with T1 considered a reference. The odds ratio (OR) was interpreted as being in the high or low-risk group based on levels of Zn, Cu, or Zn/Cu tertiles (2 or 3) (66).

## Results

### Baseline characteristics of participants

The baseline characteristics of participants are shown in Table 1. The mean age of the 437 participants was 41.1 ± 12.6 years, and 37.3% were male, while the remaining 62.7% were female. The baseline results show that the mean serum Zn level of the participants was 12.6 ± 2.0 µmol/L, and 88.6% of subjects had Zn levels within the normal range (10.1–16.83 µmol/L). On the other hand, the mean Cu levels were 18.2 ± 4.7 µmol/L, and only 16.3% of the population had elevated Cu levels (>22 µmol/L), whereas the remaining participants were within the normal range (12–22 µmol/L). With a mean BMI of 29.98 ± 6.05 kg/m^2^, 45.8% of participants were classified as obese (30 kg/m^2^) and 33.9% as overweight (25–29.9 kg/m^2^). The mean waist/hip size was 88.46 ± 14.5 cm, and 18.8% had high waist/hip size (Males: >102; Females: >88). Moreover, 32.8% of the participants had elevated fasting glucose levels (>5.5 mmol/L) with a mean value of 5.77 ± 1.89 mmol/L. The participants’ mean insulin level was 11.97 ± 16.4 µU/ml, and 94.4% fell within the normal range (3.3–5.5 µU/ml). In addition, the mean HbA1C was 5.7 ± 1.2%, and approximately 20.2% of participants had diabetes (>6.5%). Finally, around 96.1% of the subjects had C-peptide levels within the normal range (0.78–5.19 ng/ml) with a mean of 2.1 ± 1.2 ng/ml.

**Table 1:** Baseline characteristics of participants.

N = 437 Gender: Male = 37.3%; Female = 62.7% Age: Mean 41.1; SD 12.6					
Zinc level					
Mean	SD	% of low zinc <10.1	% of normal zinc 10.1–16.83	% of high zinc >16.83	
12.6 µmol/L	2.0	9.1% (*n* = 40)	88.6% (*n* = 387)	2.3% (*n* = 10)	
Copper level					
Mean	SD	% of low Cu <11	% of normal Cu 11–22	% of high Cu >22	
18.21 µmol/L	4.69	1.8% (*n* = 8)	81.9% (*n* = 358)	16.3% (*n* = 71)	
Metabolic markers					
BMI					
Mean	SD	% of Underweight <18.5	% of Normal weight 18.5–24.9	% of overweight 25–29.9	% of Obese ≥30
29.98 kg/m^2^	6.05	1.8% (*n* = 8)	18.5% (*n* = 81)	33.9% (*n* = 148)	45.8% (*n* = 200)
Hip/waist size					
Mean	SD	% of normal	% of high Highest quintile		
88.46 cm	14.5	81.2% (*n* = 355)	18.8% (*n* = 82)		
Fasting glucose					
Mean	SD	% of low <3.3	% of normal 3.3–5.5	% of high >5.5	
5.77 mmol/L	1,89	0.3% (*n* = 1)	66.9% (*n* = 292)	32.8% (*n* = 143)	
Insulin					
Mean	SD	% of low <2	% of normal 2–23	% of high >23	
11.97 µU/ml	16.40	0% (*n* = 0)	94.4% (*n* = 401)	5.6% (*n* = 24)	
HbA1c					
Mean	SD	% of low <5.7	% of normal 5.7%–6.4%	% of high ≥6.5	
5.71%	1.20	6.2% (*n* = 27)	73.6% (*n* = 321)	20.2% (*n* = 88)	
C-peptide					
Mean	SD	% of low <0.78	% of normal 0.78–5.19	% of high >5.19	
2.06 ng/ml	1.16	2.8% (*n* = 12)	96.1% (*n* = 418)	1.1% (*n* = 5)	
Cardiovascular markers					
Total cholesterol					
Mean	SD	% of normal Min to 5.2	% of high >5.2		
4.97 mmol/L	1.02	64.5% (*n* = 281)	35.5% (*n* = 155)		
HDL-cholesterol					
Mean	SD	% of Low Min to 1.55	% of Normal >1.55		
1.42 mmol/L	0.40	67.1% (*n* = 262)	32.9% (*n* = 143)		
LDL-cholesterol					
Mean	SD	% of normal Min to 2.59	% of high >2.59		
2.98 mmol/L	0.93	66.8% (*n* = 290)	33.2% (*n* = 144)		
Triglycerides					
Mean	SD	% of normal Min to 1.69	% of high >1.69		
1.28 mmol/L	0.93	78.9% (*n* = 344)	21.1% (*n* = 92)		
Systolic blood pressure (SBP)					
Mean	SD	% of Normal <120	% of elevated 120–129	% of high ≥130	
114.25 mmHg	16.09	69.6% (*n* = 304)	14.2% (*n* = 62)	16.2% (*n* = 71)	
Diastolic blood pressure (DBP)					
Mean	SD	% of normal <80	% of high ≥80		
66.82 mmHg	10.79	88.3% (*n* = 386)	11.7% (*n* = 51)		
Heart rate					
Mean	SD	% of normal 60–100	% of high >100		
70.42 beats/min	10.12	86.0% (*n* = 376)	14.0% (*n* = 61)		
Coagulation tests					
Prothrombin time (PT)					
Mean	SD	% of low <9.4	% of normal 9.4–12.5	% of high >12.5	
12.08 s	1.09	0% (*n* = 0)	74.5% (*n* = 324)	25.5% (*n* = 111)	
International normalization ratio (INR)					
Mean	SD	% of normal Min to 1.1	% of high >1.1		
1.04	0.10	93.3% (*n* = 406)	6. 7% (*n* = 29)		
Activated partial thromboplastin time (aPTT)					
Mean	SD	% of low <25.1	% of normal 25.1–36.5	% of high >36.5	
34.21 s	3.04	0% (*n* = 0)	80.0% (*n* = 348)	20.0% (*n* = 87)	
Fibrinogen					
Mean	SD	% of low <2	% of normal 2–4.1	% of high >4.1	
3.36 g/L	0.65	0.9% (*n* = 4)	88.8% (*n* = 386)	10.3% (*n* = 45)	
Cardiac markers					
Myoglobin					
Mean	SD	% of low <1 (Male and Female)	% of normal	% of high	
			Male: 1–78	Male: >78	
			Female: 1–47	Female: >47	
25.21 ng/ml	21.24	0% (*n* = 0)	95.9% (*n* = 418)	4.1% (*n* = 18)	
NT-proBNP					
Mean	SD	% of normal	% of high		
		Min to 125 (<75 years old)	>125 (<75 years old)		
		Min to 450 (≥75 years old)	>450 (≥75 years old)		
37.00 pg/ml	76.18	97.7% (*n* = 426)	2.3% (*n* = 10)		
Creatine kinase					
Mean	SD	% of low	% of normal	% of high	
		Male: < 39	Male: 39–232	Male: >232	
		Female: < 2	Female: 2–160	Female: >160	
99.20 U/L	72.72	0.7% (*n* = 2)	89.7% (*n* = 261)	9.6% (*n* = 28)	

Table 1 also summarizes the results of several cardiovascular markers. The mean TC at baseline was 5.0 ± 1.0 mmol/L, and around 35.5% of the participants had elevated TC levels (>5.2 mmol/L). Additionally, 67.1% of the patients had low HDL-C levels (1.55 mmol/L) with a mean of 1.4 ± 0.4 mmol/l. Regarding LDL-C levels, the mean LDL-C was 3.0 ± 0.9 mmol/L, and about 33.2% of the participants had elevated LDL-C (>2.59 mmol/L). The mean TG levels were 1.28 ± 0.9 mmol/L, and 21.1% of the participants had high LDL-C levels (>1.69 mmol/L). However, the mean systolic blood pressure (SBP) was 114.25 ± 16.09 mm Hg, 14.2% of participants had elevated SBP (120-129 mm Hg), and 16.2% had high SBP (>130 mm Hg). Only 11.7% of the participants had high diastolic blood pressure (≥80 mm Hg) with a mean of 66.82 ± 10.79 mm Hg. The participants’ mean heart rate was 70.42 ± 10.12 beats per minute, and only 14.0% had a high heart rate (>100 beats per minute). The coagulation tests’ results revealed that the mean Prothrombin Time (PT) was 12.1 ± 1.1 s and that 25.5% of participants had elevated PT values (>12.5 s). The mean international normalization ratio (INR) was 1.0 ± 0.1, and most participants (93.3%) a normal INR. The mean activated partial normalization ratio (aPTT) was 34.2 ± 3.0 s, and around 20% of the participants had elevated levels of aPTT (>36.5 s). The mean fibrinogen level was 3.4 ± 0.7 g/L, and most participants (88.8%) had fibrinogen levels within the normal range (2–4.1 g/L). Myoglobin, NT-proBNP, and creatine kinase were also analyzed as cardiac markers. The mean myoglobin level was 25.2 ± 21.24 ng/mL, and most participants (95.9%) were within the normal range (Males: 1–78 ng/ml; Females: 1–47 ng/ml). Moreover, the mean NT-proBNP was 37.0 ± 76.2 pg/ml, and most participants (97.7%) had NT-proBNP levels within the normal range (Male: 125 pg/ml; Female: 450 pg/ml). Finally, the mean creatine kinase level was 99.2 ± 72.7 U/L; only 9.6% of participants had elevated levels (Males: >232 U/L; Females: >160 U/L).

### Distribution of CVD risk markers based on circulating levels Zn and Cu

Tables 2–4 show the distribution of various CVD risk markers according to Zn, Cu, and Zn/Cu ratio tertiles. The study population was divided into three groups based on their levels of Zn, Cu, and Zn/Cu ratio, and the data are presented as mean values with standard deviations for each group.

**Table 2:** Distribution of CVD risk markers according to zinc tertiles.

Variables mean (SD) or *N* (%)	Zinc			*P* value
	Tertile 1 (*n* = 146) <11.63	Tertile 2 (*n* = 146) 11.63–13.32	Tertile 3 (*n* = 145) >13.32	
Age	40.79 (12.43)	41.79 (12.17)	40.65 (13.18)	NS
Gender *N* (%)				
Male	52 (35.6)	66 (45.2)	35 (31.1)	
Female	94 (64.4)	80 (54.8)	100 (68.9)	
Metabolic markers				
BMI	30.2 (6.2)	29.7 (6.2)	30.0 (5.8)	NS
Hip/waist circumference	86.7 (14.3)	88.9 (15.5)	89.8 (13.6)	NS
Glucose	5.6 (1.9)	5.9 (2.1)	5.8 (1.7)	NS
Insulin	13.8 (21.3)	10.5 (5.6)	11.6 (17.9)	NS
HbA1C	5.6 (1.1)	5.7 (1.2)	5.8 (1.2)	NS
C-peptide	2.1 (1.3)	2.1 (1.3)	1.9 (0.7)	NS
HOMA-IR	4.1 (8.0)	2.9 (2.6)	3.5 (9.2)	NS
TyG index	8.4 (0.6)	8.6 (0.7)	8.6 (0.6)	**0.008**
TG/HDL ratio	0.9 (0.7)	1.1 (1.1)	1.1 (0.7)	**0.004**
Cardiovascular markers				
Total cholesterol	4.8 (0.9)	5.1 (1.1)	5.0 (1.0)	NS
HDL-cholesterol	1.5 (0.4)	1.4 (0.4)	1.4 (0.4)	NS
LDL-cholesterol	2.9 (0.9)	3.1 (1.0)	3.0 (0.9)	NS
Triglycerides	1.1 (0.6)	1.4 (1.4)	1.3 (0.6)	**0.006**
Systolic blood pressure	112.9 (15.7)	114.9 (16.9)	115.0 (15.7)	NS
Diastolic blood pressure	65.7 (10.4)	67.9 (11.9)	66.8 (9.9)	NS
Pulse rate	71.3 (10.6)	70.4 (9.6)	69.6 (10.1)	NS
10-year ASCVD risk score	3.1 (4.0)	3.6 (5.5)	3.0 (2.7)	NS
Coagulation tests				
Prothrombin time (PT)	12.2 (1.4)	12.1 (1.0)	11.9 (0.8)	0.050
International normalization ratio (INR)	1.1 (0.1)	1.0 (0.1)	1.0 (0.1)	**0.049**
Activated partial thromboplastin time (aPTT)	34.2 (2.9)	34.1 (3.0)	34.3 (3.2)	NS
Fibrinogen	3.4 (0.7)	3.3 (0.7)	3.3 (0.6)	NS
Cardiac markers				
Myoglobin	24.6 (23.0)	24.9 (19.3)	26.1 (21.3)	NS
NT-proBNP	38.6 (37.6)	41.0 (114.4)	31.3 (54.4)	**<0.001**
Creatine kinase	145.0 (416.0)	146.2 (502.0)	127.9 (252.4)	NS
Carotid doppler (common carotid arteries)				
Pulsatility index (left)	2.0 (0.7)	2.0 (0.7)	2.0 (0.5)	NS
Pulsatility index (right)	2.0 (0.6)	2.0 (0.6)	2.0 (0.6)	NS
Resistivity index (left)	0.8 (0.1)	0.8 (0.1)	0.7 (0.1)	NS
Resistivity index (right)	0.8 (0.1)	0.8 (0.1)	0.8 (0.1)	NS
Systolic to diastolic velocity ratio (left)	4.2 (1.1)	4.4 (2.7)	4.1 (0.9)	NS
Systolic to diastolic velocity ratio (right)	4.2 (1.0)	4.4 (3.7)	4.3 (1.2)	NS
Mean intima/media thickness (left)	0.6 (0.1)	0.5 (0.1)	0.6 (0.1)	NS
Mean intima/media thickness (right)	0.5 (0.1)	0.5 (0.1)	0.5 (0.1)	NS
Viacorder test results (arterial stiffness)				
Pulse power index (PPI)	1.21 (0.15)	1.25 (0.15)	1.21 (0.24)	**0.005**
Pulse wave velocity (PWV)	12.82 (5.97)	12.03 (3.89)	12.58 (4.92)	NS

Bold numbers indicate statistically significant *P*-values.

**Table 3:** Distribution of CVD risk markers according to copper tertiles.

Variables mean ± SD or *N* (%)	Copper			*P-*value
	Tertile 1 (*n* = 147) < 15.8	Tertile 2 (*n* = 146) 15.8–19.1	Tertile 3 (*n* = 144) >19.1	
Age	39.07 (13.1)	41.97 (12.96)	42.22 (11.43)	NS
Gender				
Male	62 (42.2)	52 (35.6)	49 (34.1)	
Female	85 (57.8)	94 (64.4)	95 (65.9)	
Metabolic markers				
BMI	28.1 (5.5)	29.8 (5.7)	32.1 (6.3)	**<0.001**
Hip/waist circumference	88.2 (15.1)	88.0 (13.9)	89.2 (14.5)	NS
Glucose	5.5 (1.4)	5.8 (1.8)	6.0 (2.3)	NS
Insulin	12.5 (19.5)	10.8 (10.1)	12.7 (18.1)	NS
HbA1C	5.4 (0.8)	5.8 (1.3)	5.9 (1.4)	**<0.001**
C-peptide	2.2 (1.4)	1.9 (0.9)	2.1 (1.2)	NS
HOMA-IR	3.05 (7.1)	3.0 (3.9)	4.0 (9.4)	**0.070**
TyG index	8.5 (0.6)	8.5 (0.7)	8.6 (0.6)	NS
TG/HDL ratio	1.1 (0.8)	1.0 (0.8)	1.0 (1.0)	NS
Cardiovascular markers				
Total cholesterol	4.8 (1.0)	5.1 (1.1)	5.1 (1.0)	**0.009**
HDL-cholesterol	1.3 (0.3)	1.4 (0.4)	1.5 (0.4)	**<0.001**
LDL-cholesterol	2.9 (1.0)	3.0 (0.9)	3.0 (0.9)	NS
Triglycerides	1.2 (0.6)	1.3 (1.2)	1.3 (0.8)	NS
Systolic blood pressure	114.6 (15.0)	113.3 (16.3)	114.9 (17.0)	NS
Diastolic blood pressure	68.2 (12.0)	66.1 (9.3)	66.2 (10.9)	NS
Pulse rate	68.7 (10.2)	71.1 (9.8)	71.5 (10.2)	**0.030**
10-year ASCVD risk score	3.2 (3.2)	3.6 (5.3)	2.9 (4.0)	NS
Coagulation tests				
Prothrombin time (PT)	12.2 (1.0)	12.1 (1.4)	11.9 (0.8)	**0.025**
International normalization ratio (INR)	1.0 (0.1)	1.0 (0.1)	1.0 (0.1)	NS
Activated partial thromboplastin time (aPTT)	34.5 (3.1)	34.2 (3.2)	33.9 (2.8)	NS
Fibrinogen	3.0 (0.6)	3.4 (0.6)	3.6 (0.6)	**<0.001**
Cardiac markers				
Myoglobin	28.9 (25.5)	23.3 (16.3)	23.5 (20.6)	**<0.001**
NT-proBNP	30.6 (111.8)	35.9 (36.9)	44.4 (59.3)	**<0.001**
Creatine kinase	224.5 (621.9)	94.2 (67.0)	125.3 (407.5)	**<0.001**
Carotid doppler (common carotid arteries)				
Pulsatility index (left)	2.0 (0.8)	2.0 (0.5)	1.9 (0.5)	NS
Pulsatility index (right)	2.0 (0.6)	2.0 (0.5)	2.0 (0.6)	NS
Resistivity index (left)	0.8 (0.1)	0.8 (0.1)	0.8 (0.1)	NS
Resistivity index (right)	0.7 (0.1)	0.8 (0.1)	0.8 (0.1)	NS
Systolic to diastolic velocity ratio (left)	4.4 (2.7)	4.3 (1.1)	4.1 (0.9)	NS
Systolic to diastolic velocity ratio (right)	4.2 (1.0)	4.4 (1.2)	4.4 (3.7)	NS
Mean intima/media thickness (left)	0.5 (0.1)	0.5 (0.1)	0.6 (0.1)	NS
Mean Intima/media thickness (right)	0.5 (0.1)	0.5 (0.1)	0.5 (0.1)	NS
Viacorder test results (arterial stiffness)				
Pulse power index (PPI)	1.21 (.16)	1.23 (.24)	1.23 (.15)	NS
Pulse wave velocity (PWV)	12.27 (5.74)	12.54 (4.93)	12.63 (4.22)	NS

Bold numbers indicate statistically significant *P*-values.

**Table 4:** Distribution of CVD risk markers according to Zn/Cu ratio tertiles.

Variables	Ratio Zn/Cu			*P-*value
	Tertile 1 (*n* = 146) < 0.629	Tertile 2 (*n* = 146) 0.629–0.799	Tertile 3 (*n* = 145) >0.799	
Age	41.90 (11.36)	43.25 (12.78)	38.06 (13.05)	NS
Gender				
Male	52 (35.6)	53 (36.3)	58 (40)	
Female	94 (64.4)	93 (63.7)	87 (60)	
Metabolic markers				
BMI	31.4 (6.0)	30.3 (6.1)	28.2 (5.7)	**<0.001**
Hip/waist circumference	86.7 (13.1)	89.8 (15.4)	88.8 (14.8)	NS
Glucose	5.8 (2.0)	5.9 (2.0)	5.6 (1.6)	NS
Insulin	12.6 (19.5)	13.1 (19.6)	10.2 (6.4)	NS
HbA1C	5.8 (1.3)	5.8 (1.2)	5.5 (1.1)	**0.007**
C-peptide	2.0 (1.2)	2.1 (0.9)	2.1 (1.3)	NS
HOMA-IR	3.9 (9.6)	3.9 (7.3)	2.8 (2.9)	NS
TyG index	8.4 (0.6)	8.6 (0.7)	8.5 (0.6)	NS
TG/HDL ratio	0.9 (0.6)	1.1 (1.1)	1.1 (0.8)	**0.011**
Cardiovascular markers				
Total cholesterol	5.0 (0.9)	5.1 (1.1)	4.8 (1.0)	**0.029**
HDL-cholesterol	1.5 (0.4)	1.4 (0.4)	1.3 (0.4)	**<0.001**
LDL-cholesterol Calc	3.0 (0.8)	3.1 (1.0)	2.9 (1.0)	NS
Triglycerides	1.2 (0.6)	1.4 (1.4)	1.2 (0.6)	**0.033**
Systolic blood pressure	114.0 (16.9)	113.7 (16.7)	115.1 (14.6)	NS
Diastolic blood pressure	65.9 (10.4)	65.8 (10.7)	68.7 (11.1)	**0.034**
Pulse rate	71.7 (10.6)	70.7 (8.9)	68.8 (10.6)	**0.046**
10-year ASCVD risk score	2.9 (3.9)	3.8 (5.3)	3.0 (2.7)	NS
Coagulation tests				
Prothrombin time (PT)	12.0 (0.8)	12.1 (1.4)	12.2 (0.9)	NS
International normalization ratio (INR)	1.0 (0.1)	1.0 (0.1)	1.0 (0.1)	NS
Activated partial thromboplastin time (aPTT)	34.0 (2.8)	34.3 (2.9)	34.4 (3.4)	NS
Fibrinogen	3.6 (0.6)	3.4 (0.6)	3.0 (0.6)	**<0.001**
Cardiac markers				
Myoglobin	23.7 (23.4)	23.2 (11.8)	28.8 (25.6)	**<0.001**
NT-proBNP	44.8 (49.2)	37.5 (50.8)	28.5 (111.6)	**<0.001**
Creatine kinase	124.5 (397.9)	92.1 (43.9)	236.7 (646.8)	**<0.001**
Carotid doppler (common carotid arteries)				
Pulsatility index (left)	1.9 (0.5)	2.1 (0.7)	2.0 (0.7)	**0.042**
Pulsatility index (right)	2.0 (0.6)	2.0 (0.6)	2.0 (0.6)	NS
Resistivity index (left)	0.7 (0.1)	0.8 (0.1)	0.7 (0.1)	NS
Resistivity index (right)	0.8 (0.1)	0.8 (0.1)	0.7 (0.1)	NS
Systolic to diastolic velocity ratio (left)	4.1 (1.0)	4.6 (2.8)	4.1 (0.9)	NS
Systolic to diastolic velocity ratio (right)	4.4 (3.6)	4.4 (1.2)	4.2 (1.2)	NS
Mean Intima/media thickness (left)	0.6 (0.1)	0.5 (0.1)	0.5 (0.1)	**0.034**
Mean Intima/media thickness (right)	0.5 (0.1)	0.5 (0.1)	0.5 (0.1)	NS
Viacorder test results (arterial stiffness)				NS
Pulse power index (PPI)	1.23 (.16)	1.21 (.14)	1.23 (.29)	NS
Pulse wave velocity (PWV)	12.89 (4.81)	12.15 (5.34)	12.39 (4.83)	NS

Bold numbers indicate statistically significant *P*-values.

Table 2 compares serum Zn levels by tertile to markers of CVD. Metabolic markers such as BMI, hip/waist circumference, glucose, insulin, HbA1C, C-peptide, and HOMA-IR showed no significant differences among the three zinc tertiles (*p* > 0.05). However, the TyG index and TG/HDL ratio differed significantly among the tertiles (*p* = 0.008 and *p* = 0.004, respectively). Regarding cardiovascular markers, TG levels were found to be significantly different among the Zn tertiles (*p* = 0.006). In contrast, TC, HDL-C, LDL-C, systolic and diastolic blood pressure, pulse rate, and the 10-year ASCVD risk score showed no significant differences (*p* > 0.05). Coagulation test results showed that the aPTT and fibrinogen did not differ significantly among the Zn tertiles (*p* > 0.05). While INR and PT had a *p*-value of 0.049 and 0.05, respectively. The cardiac markers myoglobin and creatine kinase showed no significant differences among the tertiles (*p* > 0.05), while NT-proBNP showed a significant difference (*p* < 0.001) among tertiles with levels in third tertile (T3) being lower than T2 and T1. The results of carotid Doppler analysis of common carotid arteries showed no significant differences between the Zn tertiles. Regarding markers of arterial stiffness, PPI was higher in Zn T2 compared to T1 (*p* = 0.005); however, no difference was observed for PWV. These results suggest that Zn levels may play a role in modulating some CVD risk markers, such as the TyG index, TG/HDL ratio, and TG levels.

Table 3 presents the results of comparing markers of CVD risk with serum Cu levels distributed into tertiles. The key findings in Table 3 show that Cu tertiles are significantly associated with many CVD risk markers. The metabolic markers such as BMI and HbA1C significantly increased across Cu tertiles (*p* < 0.001), indicating an association between higher Cu levels and metabolic dysfunction. The cardiovascular markers such as TC (*p* = 0.009), HDL-C (*p* < 0.001), and pulse rate (*p* = 0.03) were also significantly increased with Cu tertiles. The coagulation markers such as PT and fibrinogen also significantly differed across Cu tertiles (*p* = 0.025 and *p* < 0.001, respectively). Cardiac markers such as myoglobin, NT-proBNP, and creatine kinase were also significantly associated with Cu tertiles (*p* < 0.001). Myoglobin levels decreased with higher Cu tertiles (T2 and T3) compared to T1 (*p* < 0.001). NT-proBNP levels significantly increased with Cu tertiles (*p* < 0.001). However, creatine kinase levels decreased with higher Cu tertiles (T2 and T3) compared to T1 (*p* < 0.001).

It is noteworthy that there was no statistically significant correlation between the tertiles of Cu and various metabolic and cardiovascular markers, including glucose, insulin, C-peptide, TyG index, TG/HDL ratio, LDL-C, TG, systolic and diastolic blood pressure, INR, aPTT, and PI in the carotid doppler (*p* > 0.05).

The presented findings indicate a substantial correlation between elevated levels of Cu and negative outcomes related to metabolism, cardiovascular health, coagulation, and cardiac function indicators. The results suggest that high Cu levels may have a significant impact on the progression of CVD.

Table 4 presents a comparison between tertiles of the Zn/Cu ratio and risk factors for CVD. The findings, as presented in Table 4, indicate that there is a statistically significant difference in various parameters across the three tertiles of Zn/Cu ratio. These parameters include BMI (*p* < 0.001), HbA1C (*p* = 0.007), HDL-C (*p* < 0.001), TC (*p* = 0.029), TG (*p* = 0.033), TG/HDL ratio (*p* = 0.011), DBP (*p* = 0.034), pulse rate (*p* = 0.046), fibrinogen (*p* < 0.001), myoglobin (*p* < 0.001), NT-proBNP (*p* < 0.001), and creatine kinase (*p* < 0.001).

The study revealed a noteworthy finding indicating that individuals with greater Zn/Cu ratios (T3), had lower values for various health indicators including BMI, HbA1C, TC, TG, HDL-C, pulse rate, and cardiac markers such as myoglobin and NT-proBNP, in comparison to those in T1. However, participants in the highest tertile exhibited elevated levels of TG/HDL ratio, fibrinogen, cardiac marker creatine kinase, and DBP in comparison to those in the lowest tertile.

Furthermore, the study revealed a substantial disparity in the PI inside the left common carotid artery among the three tertiles of Zn/Cu ratio (*p* = 0.042). Specifically, individuals in the highest tertiles had a notably higher PI value in comparison to those in the lowest tertile (T1).

In summary, these findings indicate that an elevated Zn/Cu ratio is linked to a more advantageous profile of CVD risk markers. This includes a lower body BMI, enhanced glycemic management, improved lipid profile, reduced inflammation, and better cardiac function. Nevertheless, individuals belonging to the uppermost tertile exhibited elevated DBP and creatine kinase levels, indicating a plausible impact on cardiovascular performance. The findings additionally indicate a potential association between the PI in the left common carotid artery and the Zn/Cu ratio, suggesting a potential involvement of this ratio in the modulation of vascular function.

### Association between Zn, Cu, Zn/Cu ratio, and CVD risk factors: results of bivariate multinomial logistic regression

Tables 5–7 depict the multinomial logistic regression analysis that has been corrected for gender, nationality, age, and education variables. These tables specifically pertain to the variables Zn, Cu, and the Zn/Cu ratio. T1 served as the reference for all the aforementioned tables. Within the provided tables, we identify the odds risk (OR) associated with belonging to either the high or low group, as determined by the levels of Zn, Cu, or Zn/Cu tertiles (2 or 3).

**Table 5:** Association between Zn tertiles and CVD risk markers (adjusted for gender, nationality, age, and education).

Variables	Zinc					
	Tertile 2			Tertile 3		
	AOR	95% CI	*p*-value	AOR	95% CI	*p*-value
Metabolic markers						
High BMI	0.616	0.318–1.194	NS	0.655	0.341–1.257	NS
High hip/waist Circumference	1.417	0.759–2.644	NS	1.197	0.625–2.291	NS
High glucose	1.334	0.758–2.348	NS	1.488	0.837–2.643	NS
High insulin	.669	0.251–1.778	NS	0.340	0.102–1.125	NS
High HbA1c	2.514	1.194–5.292	**0.015**	3.207	1.535–6.697	**0.002**
High C-peptide	1.030	0.125–8.464	NS	–	–	–
High HOMA-IR	0.843	0.504–1.408	NS	0.794	0.471–1.336	NS
High TyG index	2.207	1.285–3.791	**0.004**	2.676	1.544–4.638	**<0.001**
Cardiovascular markers						
High Total cholesterol	1.821	1.108–2.994	**0.018**	1.581	0.956–2.615	NS
Low HDL-cholesterol	0.920	0.564–1.501	NS	.746	0.454–1.227	NS
High LDL-cholesterol	1.375	0.835–2.265	NS	1.317	0.797–2.176	NS
High triglycerides	2.177	1.166–4.065	**0.015**	1.884	0.989–3.587	0.054
High systolic blood pressure	1.303	0.724–2.345	NS	1.423	0.780–2.596	NS
High diastolic blood pressure	1.980	0.954–4.109	NS	0.928	0.403–2.136	NS
High pulse rate	0.646	0.329–1.266	NS	0.662	0.341–1.285	NS
High 10-year ASCVD risk score	5.047	0.052–403.21	NS	0.003	0.0006–6.616	NS
Coagulation tests						
High prothrombin time (PT)	0.614	0.359–1.052	NS	0.374	0.210–0.666	**0.001**
High international normalization ratio (INR)	0.869	0.340–2.216	NS	0.815	0.319–2.080	NS
High activated partial thromboplastin time (aPTT)	1.215	0.685–2.155	NS	0.851	0.470–1.542	NS
High fibrinogen	0.943	0.445–2.000	NS	.537	0.229–1.256	NS
Cardiac markers						
High myoglobin	2.058	0.561–7.541	NS	1.627	0.435–6.077	NS
High NT-proBNP	0.992	0.177–5.558	NS	1.417	0.294–6.836	NS
High creatine kinase	1.323	0.509–3.440	NS	1.618	0.600–4.360	NS
Carotid doppler (common carotid arteries)						
Pulsatility index (left)	1.171	0.572–2.395	NS	1.732	0.851–3.523	NS
Pulsatility index (right)	1.130	0.555–2.303	NS	1.517	0.757–3.042	NS
Resistivity index (left)	.964	.489–1.898	NS	0.854	0.416–1.756	NS
Resistivity index (right)	1.046	0.469–2.336	NS	1.900	0.884–4.084	NS
Systolic to diastolic Velocity ratio (left)	1.103	0.568–2.144	NS	.883	0.436–1.785	NS
Systolic to diastolic Velocity ratio (right)	0.998	0.484–2.056	NS	1.598	0.807–3.164	NS
Mean intima/media Thickness (left)	0.679	0.341–1.350	NS	1.271	0.673–2.401	NS
Mean intima/media Thickness (right)	0.705	0.365–1.362	NS	.768	0.397–1.485	NS
Viacorder test results (arterial stiffness)						
Pulse power index (PPI)	2.18	0.998–4.764	0.05	1.495	0.641–3.482	NS

Bold numbers indicate statistically significant *P*-values.

**Table 6:** Association between copper tertiles and CVD risk markers (adjusted for gender, nationality, age, and education).

Variables	Copper Tertile 2			Copper Tertile 3		
	AOR	95% CI	*p*-value	AOR	95% CI	*p*-value
Metabolic markers						
High BMI	1.463	0.800–2.676	NS	2.971	1.485–5.942	**0.002**
High hip/waist Circumference	0.790	0.425–1.468	NS	0.713	0.380–1.340	NS
High glucose	0.969	0.542–1.731	NS	1.312	0.745–2.312	NS
High insulin	1.014	0.345–2.976	NS	1.084	0.370–3.176	NS
High HbA1c	1.227	0.592–2.540	NS	1.853	0.920–3.734	NS
High C-peptide	0.410	0.034–4.902	NS	–	–	–
High high HOMA-IR	0.626	0.367–1.067	NS	0.917	0.549–1.532	NS
TyG index	0.714	0.419–1.218	NS	1.049	0.624–1.764	NS
Cardiovascular markers						
High total Cholesterol	1.292	0.785–2.128	NS	1.563	0.952–2.566	NS
low HDL-cholesterol	1.697	1.010–2.851	**0.046**	2.272	1.359–3.798	**0.002**
High LDL-cholesterol	1.358	0.827–2.228	NS	1.130	0.681–1.875	NS
High triglycerides	0.609	0.340–1.093	NS	0.440	0.237–0.816	**0.009**
High systolic blood pressure	0.547		NS	0.911		NS
High diastolic blood pressure	0.417	0.196–0.889	**0.024**	0.477	0.228–0.996	**0.049**
High pulse rate	1.437	0.741–2.788	NS	0.878	0.427–1.803	NS
10-year ASCVD risk score	0.642	0.039–10.501	NS	1.567	0.048–50.692	NS
Coagulation tests						
High prothrombin time (PT)	0.823	0.479–1.411	NS	0.648	0.368–1.139	NS
High international normalization ratio (INR)	0.981	0.412–2.331	NS	0.471	0.159–1.395	NS
High activated partial thromboplastin time (aPTT)	1.239	0.695–2.206	NS	1.005	0.552–1.828	NS
High fibrinogen	3.11	1.083–8.959	**0.035**	5.039	1.812–14.007	**0.002**
Cardiac markers						
High myoglobin	1.236	0.369–4.137	NS	0.909	0.248–3.326	NS
High NT-proBNP	0.183	0.019–1.768	NS	1.095	0.261–4.583	NS
High creatine kinase	0.439	0.172–1.123	**NS**	0.270	0.095–0.765	**0.014**
Carotid doppler (common carotid arteries)						
Pulsatility index (left)	0.760	0.387–1.493	NS	0.554	0.273–1.124	NS
Pulsatility index (right)	1.697	0.857–3.360	NS	1.067	0.523–2.175	NS
Resistivity index (left)	1.014	0.511–2.015	NS	0.855	0.422–1.730	NS
Resistivity index (right)	1.902	0.849–4.257	NS	2.163	0.979–4.777	NS
Systolic to diastolic velocity ratio (left)	.847	0.438–1.636	NS	0.586	0.290–1.180	NS
Systolic to diastolic velocity ratio (right)	1.377	0.682–2.781	NS	1.339	0.671–2.673	NS
Mean intima/media thickness (left)	1.043	0.528–2.060	NS	1.399	0.732–2.673	NS
Mean intima/media thickness (right)	1.159	0.602–2.229	NS	.873	0.445–1.711	NS
Viacorder test results (arterial stiffness)						
Pulse power index (PPI)	1.834	0.835–4.026	NS	1.797	0.803–4.019	NS

Bold numbers indicate statistically significant *P*-values.

**Table 7:** Association between Zn/Cu ration and CVD risk markers (adjusted for gender, nationality, age, and education).

Variables	Zn/Cu ratio Tertile 2			Zn/Cu ratio Tertile 3		
	AOR	95% CI	*p*-value	AOR	95% CI	*p*-value
Metabolic markers						
high bmi	0.717	0.352–1.459	NS	0.404	0.211–.775	**0.006**
High hip/waist circumference	2.051	1.043–4.033	**0.037**	2.667	1.340–5.307	**0.005**
High glucose	1.391	0.800–2.416	NS	1.134	0.632–2.032	0.672
High insulin	1.400	0.510–3.841	NS	0.600	0.167–2.157	0.434
High HbA1c	1.110	0.574–2.148	NS	1.118	0.556–2.251	0.753
High C-peptide	0.469	0.049–4.432	NS	–	–	–
High HOMA-IR	1.254	0.747–2.106	NS	1.238	0.729–2.103	0.428
High TyG index	1.029	0.608–1.741	NS	1.689	0.991–2.879	0.054
Cardiovascular markers						
High total cholesterol	1.416	0.871–2.303	NS	1.054	0.638–1.743	0.835
Low HDL-cholesterol	0.752	0.464–1.220	NS	0.424	0.253–0.712	**0.001**
High LDL-cholesterol	1.367	0.828–2.255	NS	1.287	0.776–2.134	0.328
High triglycerides	1.662	0.886–3.116	NS	2.233	1.185–4.207	**0.013**
High systolic blood pressure	0.898	0.497–1.622	NS	1.596	0.874–2.913	NS
High diastolic blood pressure	0.776	0.338–1.781	NS	1.912	0.925–3.955	NS
High pulse rate	0.943	0.483–1.842	NS	0.954	0.482–1.888	NS
10-year ASCVD risk score	0.681	0.026–17.475	NS	1.392	0.055–35.294	NS
Coagulation tests						
High prothrombin time (PT)	0.963	0.551–1.683	NS	0.831	0.476–1.450	NS
High international normalization ratio (INR)	1.852	0.651–5.267	NS	1.577	0.558–4.458	NS
High activated partial thromboplastin time (aPTT)	0.996	0.558–1.778	NS	0.887	0.492–1.597	NS
High fibrinogen	0.329	0.150–0.720	**0.005**	0.273	0.110–0.676	**0.005**
Cardiac markers						
high myoglobin	1.094	0.308–3.884	NS	1.427	0.405–5.025	NS
High NT-proBNP	1.354	0.294–6.231	NS	0.921	0.141–6.000	NS
High creatine kinase	1.538	1.004–5.499	NS	4.627	2.118–10.687	**0.004**
Carotid doppler (common carotid arteries)						
Pulsatility index (left)	1.502	0.726–3.108	NS	1.562	0.769–3.173	NS
Pulsatility index (right)	1.555	0.777–3.111	NS	1.041	0.513–2.112	NS
Resistivity index (left)	1.784	0.890–3.574	NS	1.019	0.483–2.149	NS
Resistivity index (right)	1.175	0.564–2.444	NS	0.826	0.380–1.796	NS
Systolic to diastolic velocity ratio (left)	2.123	1.037–4.344	**0.039**	1.706	0.822–3.536	NS
Systolic to diastolic velocity ratio (right)	1.817	0.918–3.599	NS	1.058	0.512–2.186	NS
Mean intima/media thickness (left)	0.389	0.192–0.788	**0.009**	0.778	0.416–1.455	NS
Mean intima/media thickness (right)	0.693	0.347–1.387	NS	1.232	0.644–2.356	NS
Viacorder test results (arterial stiffness)						
Pulse power index (PPI)	0.867	0.409–1.838	NS	0.759	0.355–1.624	NS

Bold numbers indicate statistically significant *P*-values.

The association between Zn tertiles and CVD risk markers was investigated using multinomial logistic regression analysis, with adjustments made for gender, nationality, age, and education. The findings of this study are presented in Table 5. The study revealed that there was no statistically significant correlation between zinc levels and metabolic indicators, such as BMI, hip/waist circumference, glucose levels, insulin levels, C-peptide levels, and HOMA-IR. Nevertheless, the study revealed noteworthy positive correlations between Zn levels and HbA1C, with ORs of 2.51 (*p* = 0.015) and 3.2 (*p* = 0.002) for T2 and T3, respectively. There was a positive association between Zn levels and the TyG index, with ORs of 2.207 (*p* = 0.004) and 2.67 (*p* < 0.001) for T2 and T3, respectively.

In relation to cardiovascular indicators, the study observed a noteworthy and favorable correlation between Zn levels and TC. The OR for the middle tertile was determined to be 1.8 (*p* = 0.018). Furthermore, a significant correlation was found between Zn levels and TG levels, with ORs of 2.17 (*p* = 0.015) for the medium tertile. There were no statistically significant correlations observed between Zn levels and systolic or diastolic blood pressures, pulse rate, or the 10-year ASCVD risk score.

In relation to coagulation tests, there was no significant correlation observed between Zn levels and INR, aPTT, or fibrinogen levels. Nevertheless, a significant inverse correlation was identified between Zn levels and PT, as evidenced by an OR of 0.37 (*p* = 0.001) for the uppermost tertile (T3).

The study also investigated cardiac markers and found no significant associations between Zn levels and myoglobin, NT-proBNP, or creatine kinase levels. The results of 3D carotid doppler analysis of common carotid arteries and Viacorder examination of arterial stiffness were also included in Table 5. No significant associations were found between Zn levels and any of the tested parameters in carotid Doppler or viacorder examinations.

The results of the multinomial logistic regression analysis examining the association between Cu tertiles and CVD risk markers are presented in Table 6. The analysis was adjusted for gender, nationality, age, and education. Regarding the metabolic markers, compared to the reference group (T1), individuals in T3 had significantly higher ORs for high BMI (OR = 2.79, *p* = 0.002), while no significant associations were found with hip/waist circumference, glucose, insulin, HbA1C, C-peptide, HOMA-IR, TyG index, and TG/HDL ratio.

Among CVD markers, individuals in T2 and T3 had significantly higher ORs for high HDL-C levels (OR = 1.7, *p* = 0.046, and OR = 2.3, *p* = 0.002, respectively) compared to individuals in the lowest tertile. Individuals in T3, on the other hand, had lower ORs for high TG levels (OR = 0.4, *p* = 0.009) compared to the reference group. However, no significant associations were found between Cu tertiles and TC or LDL-C levels. With regards to blood pressure, only diastolic blood pressure was significantly correlated with Cu levels. Participants in T2 and T3 had a lower OR for high DBP (OR = 0.41, *p* = 0.024, and OR = 0.47, *p* = 0.049, respectively) compared to the reference group (T1).

In terms of coagulation and cardiac markers, compared to the reference group (T1), individuals in T2 and T3 had significantly higher ORs for high fibrinogen levels (OR = 3.1, *p* = 0.035; OR = 5.03, *p* = 0.002, respectively), while no significant associations were found with PT, INR, aPTT, myoglobin, and NT-proBNP. However, participants in the highest tertile had a significantly low OR for high creatinine kinase levels (OR = 0.27, *p* = 0.014) compared to T1. No significant associations were found between Cu tertiles and carotid Doppler (common carotid arteries) or viacorder examination results.

The association of the Zn/Cu ratio tertiles with CVD risk markers was also investigated using bivariate multinomial logistic regression. The results are presented in Table 7. The study's key findings are that participants in the highest tertile of the Zn/Cu ratio had a significantly higher risk for several CVD risk markers than those in the lowest tertile (T1). Specifically, participants in the highest tertile had significantly a lower odd ratio for high BMI (OR = 0.4, *p* = 0.006). At the same time, participants in the highest tertiles T2 and T3 had a significantly higher risk for high hip/waist circumference (OR = 2.05, *p* = 0.037; OR = 2.66, *p* = 0.005, respectively). Individuals in the highest tertile (T3) had a significantly high OR for high TG levels (OR = 2.23, *p* = 0.013).

Additionally, a negative association between the highest Zn/Cu tertile (T3) and high HDL-C levels was observed (OR = 0.42, *p* = 0.001). Regarding cardiac function indicators, a significant positive correlation was observed between the uppermost tertile and OR of elevated creatine kinase levels (OR = 4.62, *p* = 0.004) in comparison to T1. It is noteworthy that in the context of coagulation tests, there exists a negative association between middle and high tertiles (T2 and T3) and fibrinogen levels (OR = 0.33, *p* = 0.005; OR = 0.27, *p* = 0.005, respectively). Furthermore, it was shown that individuals in the middle tertile (T2) had a notably increased likelihood of high SDVR in the left common carotid artery (OR = 2.12, *p* = 0.039). No statistically significant relationships were found between the tertiles of the Zn/Cu ratio and other risk markers for CVD, such as measures of arterial stiffness and mean intima-media thickness. Overall, the study indicates that an elevated Zn/Cu ratio could potentially be linked to heightened CVD risk indicators.

## Discussion

Optimal circulating levels of Zn and Cu are crucial for the proper functioning and structural integrity of various cellular functions. The primary objective of the present study was to examine the correlation between the blood levels of Zn and Cu, as well as their ratio, with indicators of CVD risk in a carefully characterized population from Qatar. This study is the inaugural investigation of this relationship within the population in the state of Qatar. The results show that 88.6% of the participants exhibited Zn levels that fell within the established normal range while 9.3% of the study cohort showed a deficiency in Zn, and 2.3% exhibited excessive levels of Zn. The results also revealed that 16.3% of the population exhibited excessive levels of Cu, while the majority of participants (81.9%), were within the normal range and only 1.8% of individuals were deficient in Cu. The study population was stratified into tertiles according to their Zn, Cu, and Zn/Cu ratio levels, and relationships were examined through a bivariate analysis, followed by a multinomial logistic regression. Individuals in the highest tertiles of Zn levels had an increased likelihood of experiencing unfavorable metabolic functions, including elevated levels of TG, TG/HDL ratio, and TyG index. The same individuals also showed considerably reduced ORs for prolonged PT. Elevated levels of Cu were found to have a positive correlation with increased levels of HDL-C and TC. In contrast, they decreased the chances of high creatinine kinase and prolonged prothrombin PT.

The results of the study showed that individuals with higher Zn levels (T2 and T3) were more likely to have high levels of HbA1C, TC, and TG compared to those in T1. Specifically, individuals with Zn levels in T2 were 2.5 times more likely to have high HbA1C levels, 1.8 times more likely to have high TC, and 2.1 times more likely to have high TG than those in T1. Those with Zn levels of T3 were 3.2 times more likely to have high HbA1C. However, they were 63% less likely to have a prolonged PT. The positive association between Zn levels and HbA1C observed in this study suggests that Zn levels may play a role in the development of insulin resistance and dyslipidemia, which is consistent with previous research (67). Paradoxically, a study by Bandeira et al. evaluated the relationship between the Zn-related nutritional status and glycemic and insulinemic markers in individuals with type 2 diabetes and found a negative association between Zn plasma levels and HbA1C (68). While the present study revealed a significant positive correlation between Zn levels and TC and TG levels, previous research indicated that Zn supplementation may have a positive impact on plasma lipid profile (69). In addition, a meta-analysis that analyzed randomized controlled trials found that patients with hyperlipidemia who received Zn supplementation experienced a significant decrease in their TC and TG levels (70). Previous studies regarding the relationship between Zn and CVD risk markers were inconsistent. For example, some studies associated Zn deficiency with increased TC and LDL-C levels (42), while other reports found no significant association between Zn levels and lipid profile (43), fatty acids (44), and CVD risk (45, 46). Ruiz-Canela et al. reported that higher serum Zn levels were associated with a lower risk of incident CVD events in a population of older adults (71). Nevertheless, there have been contradictory findings from other published reports showing that there is no substantial correlation between Zn levels and risk markers for CVD (72). There were no statistically significant correlations observed between Zn levels and systolic and diastolic blood pressures, pulse rate, or the 10-year ASCVD risk score. A comprehensive analysis of randomized controlled studies revealed that the supplementation of Zn was associated with a considerable reduction in SBP among adults, but no discernible effect was observed on DBP (73). In relation to coagulation tests, there was no significant correlation observed between Zn levels and INR, aPTT, or fibrinogen levels. Nevertheless, a significant inverse correlation was identified between Zn concentrations in the uppermost tertile and prolonged PT. This implies that Zn levels might exert a protective effect on the process of blood coagulation. The results of this study align with a previous investigation that documented an inverse correlation between Zn levels and extended PT (73).

Participants in the uppermost tertiles of Cu levels had notably elevated ORs for high BMI and fibrinogen levels in the second and third tertiles, while demonstrating considerably reduced ORs for high TG levels in the third tertile (T3) compared to the reference group. Additionally, they exhibited an increased likelihood of possessing elevated levels of HDL-C. The observed positive correlation between Cu levels and BMI within the highest tertile suggests a potential link between elevated Cu levels and an increased risk of obesity. This finding aligns with a prior investigation that documented a direct correlation between levels of Cu blood levels and BMI, as well as the hormone leptin and the leptin/BMI ratio (74). The study revealed a favorable correlation between Cu levels and HDL-C levels in the middle and highest tertiles, with respect to cardiovascular indicators. This aligns with prior studies that have established a correlation between greater Cu tertiles and elevated levels of HDL-C (47). On the other hand, there was an inverse relationship observed between Cu levels and elevated TG levels within the uppermost tertile. This implies that increased Cu levels can have a protective effect against rising TG levels. Previous studies have demonstrated a favorable correlation between plasma Cu levels and increased levels of TC, LDL-C, and HDL-C (47). Regarding blood pressure, the present study showed that individuals in the middle and highest tertiles exhibited decreased ORs for increased DBP when compared to the reference group. This aligns with a recent study that has shown that the risk of developing hypertension is considerably reduced by consuming higher amounts of dietary Cu (<1.57 mg/day). When the consumption of Cu surpasses 1.57 mg per day, however, there is an elevated likelihood of developing hypertension, suggesting a U-shaped relationship between Cu levels and the risk of hypertension (75). This observation is supported by prior research which has reported that Cu levels exceeding 130 g/dl may increase the likelihood of developing hypertension by a factor of 1.99 (48). Regarding coagulation and cardiac indicators, the present investigation revealed that participants in the middle and highest tertiles exhibited notably elevated ORs for high fibrinogen levels. Conversely, individuals in the highest tertile had considerably reduced ORs for high creatinine kinase levels. The results of this study align with other research that has established a favorable correlation between Cu levels and fibrinogen levels (76). Nevertheless, the study could not identify any statistically significant correlations between Cu levels and PT, INR, aPTT, myoglobin, and NT-proBNP.

The analysis of the Zn/Cu ratio tertiles in relation to markers of CVD risk demonstrated that participants in the highest tertile had a significantly greater risk for multiple CVD risk markers compared to those in the lowest tertile (T1). The study findings indicate that individuals in the highest tertile T3 exhibited a statistically significant increase in the likelihood of having elevated hip/waist circumference and BMI. Participants in the uppermost tertile (T3) exhibited a notably elevated OR for elevated TG levels. Furthermore, an inverse correlation was found between the greatest tertile of Zn/Cu ratio and elevated levels of HDL-C. In relation to cardiac indicators, a robust positive correlation was seen between the uppermost tertile and OR of elevated creatinine kinase levels. Creatinine kinase serves as an indicator of myocardial injury, and its increase is linked to unfavorable cardiovascular consequences (77). This finding contradicts the outcomes of a previous investigation, which established an inverse correlation between the Zn/Cu ratio and cardiac troponin I, another indicator of heart injury (78). Therefore, further research is needed to clarify the nature of the association between the Zn/Cu ratio and cardiac markers. Interestingly, for coagulation tests, middle and high tertiles (T2 and T3) were negatively associated with fibrinogen levels. The ratio of Zn to Cu, which indicates the interaction between these two trace elements, is a stronger predictor of many pathologies than individual Zn and Cu levels (51). CVD-related mortality has been associated with a high circulating Cu/Zn ratio (52, 53). Several inflammatory indicators and serum albumin concentrations were shown to be correlated with the circulating Cu/Zn ratio. Moreover, this ratio predicted mortality in those over the age of 70 for a period of 3.5 years (22). The plasma Cu/Zn ratio was shown to be higher in individuals with stable CVD compared to those without, mostly owing to increased circulating Cu levels. However, a progressive decline in plasma Zn levels was suspected to be the primary driver of the Cu/Zn variations seen with advancing age. The Cu/Zn ratio is an important clinical biomarker and a predictor of all-cause death in those older than 70 years old. Qatar has seen a rise in obesity and non-communicable diseases due to dietary and lifestyle changes (3). Therefore, it is essential to understand the causes associated with the increase in cardiovascular risk factors. However, no previous research has been conducted in Qatar on the relationship of trace mineral levels (Zn and Cu) with the risk of CVD in the adult population.

Bivariate regression analysis findings from our study indicate that there exists an intricate correlation between Zn, Cu, their ratio, and risk factors related to CVD. Specifically, the study found that higher levels of Zn were associated with a lower risk of prolonged PT and a higher risk for high HbA1C. This finding aligns with other research that has documented a negative correlation between Zn levels and HbA1C, a biomarker indicating glycemic management in individuals with diabetes (67). Moreover, it has been demonstrated that Zn plays a pivotal role in the process of coagulation cascade, and a shortage in Zn has been linked to a heightened susceptibility to bleeding (79, 80). It has been demonstrated that the depletion of Zn can result in the inactivation of thrombin, hence causing disruptions in blood coagulation and subsequent bleeding. The modulation of coagulation can potentially be influenced by histidine-rich glycoprotein (HRG), which competes with thrombin in order to bind to the gamma chain of fibrin. The HRG protein is present in the bloodstream in conjunction with fibrinogen, and this association remains intact even after fibrin is formed. HRG exhibits the capacity to form complexes with fibrinogen and various plasma proteins, such as plasminogen, which plays a critical role in the process of fibrinolysis. This binding interaction is reliant on the presence of Zn. The structure and function of HRG can be altered synergistically by an increase in the quantity of Zn coupled with a decrease in pH (81, 82). In contrast, our study revealed a positive correlation between elevated levels of Cu and increased BMI, as well as heightened fibrinogen levels. This observation aligns with prior research that has identified a significant correlation between Cu levels and obesity (74). Furthermore, previous studies have demonstrated that Cu may induce the production of fibrinogen in the liver, leading to an elevation in its plasma concentration (83). Elevated levels of fibrinogen are recognized as a risk factor for CVD and have been linked to an increased occurrence of thrombotic events such as myocardial infarction and stroke (84). The current study revealed a significant correlation between an elevated Zn/Cu ratio and reduced levels of fibrinogen. These findings imply a plausible protective mechanism against CVD. This observation aligns with a recent study that has documented a negative correlation between the Zn/Cu ratio and the likelihood of developing heart failure (85). In a recent cohort study comprising 11,470 adults in China, researchers observed an L-shaped relationship between dietary Zn intake and the likelihood of developing CVD. This finding suggests that a moderate enhancement in dietary Zn intake could potentially be advantageous for the prevention of CVD. However, caution should be exercised to avoid excessive Zn intake (86). The present investigation did not evaluate the consumption of Zn and Cu among the study subjects. Furthermore, a significant proportion of the study participants (88.6% for Zn and 81.9% for Cu) had normal levels for both minerals, and only 2% of participants displayed deficiency. On the other hand, 9.3% and 16.3% of the participants had elevated levels of Zn and Cu, respectively. This distribution of Zn and Cu levels among participants may have hindered the detection of an L-shaped relationship between CVD risk markers and mineral serum status, and a larger cohort may be needed to investigate the effects of high and deficient zinc and copper levels on CVD risk factors among the population in Qatar.

Our study was characterized by several strengths including the large sample size, a comprehensive set of CVD risk markers, and the robust analytical approach underpinned by using tertiles to categorize Zn, Cu, and Zn/Cu ratio levels. Nonetheless, the study has some limitations that need to be considered. Firstly, the study is a retrospective observational study, and thus, it cannot establish a cause-and-effect or temporal relationship between Zn, Cu, Zn/Cu, and CVD risk factors. Secondly, the study population was focused on a specific geographic area which could limit the generalizability of the findings to other populations. Thirdly, we did not measure the dietary intake of Zn or Cu, which could have influenced our results and the patterns reported above. Fourthly, the study did not investigate the mechanism underlying the observed associations which warrants further investigations into the underlying biological pathways.

In conclusion, our study provides evidence for a complex association between Cu, Zn, and Zn/Cu ratio with CVD risk markers in the population of the state of Qatar. Notably, higher Zn levels were associated with adverse metabolic functions (e.g., HB1AC, TG levels, TG/HDL, and TyG index) and a lower risk of prolonged PT. Conversely, higher Cu levels were postpositively associated with high HDL-C levels, and a lower risk for high TG, blood pressure, creatinine kinase, and prolonged PT. A higher Zn/Cu ratio is associated with a reduced risk for prolonged PT.

A significant association was observed between a higher ratio of Zn/Cu and a decreased likelihood of extended PT, suggesting a diminished vulnerability to CVD. The aforementioned results emphasize the potential significance of maintaining adequate levels of Zn and Cu in order to reduce the risk of CVD within the population of Qatar.

In order to further explore the practical implications of these findings, it is imperative to contemplate the potential mechanisms underlying these correlations. Potential areas for future research could center on elucidating the complex biological mechanisms by which Cu and Zn exert an influence on cardiovascular health. Furthermore, conducting an investigation into the viability of incorporating dietary guidelines or interventions aimed at maintaining appropriate Zn and Cu levels could provide pragmatic approaches for managing CVD risks within this specific group.

Although our study has provided useful insights, further research is needed to validate our findings and gain a more comprehensive understanding of the underlying molecular mechanisms. To establish causality and elucidate the temporal relationship between Cu, Zn, the Zn/Cu ratio, and CVD risk in the context of the Qatari population, prospective studies are warranted. These future investigations hold the potential to inform targeted interventions and public health strategies and guidelines aimed at reducing CVD risk in this specific population.

## Data Availability

The original contributions presented in the study are included in the article, further inquiries can be directed to the corresponding authors.

## References

[1] Badimon L Chagas P Chiva-Blanch G. Diet and cardiovascular disease: effects of foods and nutrients in classical and emerging cardiovascular risk factors. Curr Med Chem. (2019) 26(19):3639–51. 10.2174/0929867324666170428103206 28462707

[2] Tsao CW Aday AW Almarzooq ZI Anderson CA Arora P Avery CL Heart disease and stroke statistics—2023 update: a report from the American heart association. Circulation. (2023) 147(8):e93–e621. 10.1161/CIR.0000000000001052 36695182 PMC12135016

[3] Kerkadi A Alkudsi DS Hamad S Alkeldi HM Salih R Agouni A. The association between zinc and copper circulating levels and cardiometabolic risk factors in adults: a study of Qatar biobank data. Nutrients. (2021) 13(8):2729. 10.3390/nu13082729 34444889 PMC8398315

[4] Leiter LA Fitchett DH Gilbert RE Gupta M Mancini GJ McFarlane PA Cardiometabolic risk in Canada: a detailed analysis and position paper by the cardiometabolic risk working group. Can J Cardiol. (2011) 27(2):e1–e33. 10.1016/j.cjca.2010.12.054 21459257

[5] Shehab A Bhagavathula A. P5317 prevalence of cardiovascular diseases in the middle-east: systemic review and meta-analysis. Eur Heart J. (2019) 40(Supplement_1):ehz746.0287. 10.1093/eurheartj/ehz746.0287

[6] Syed MA Alnuaimi AS Zainel AJ Qotba/A HA. Prevalence of non-communicable diseases by age, gender and nationality in publicly funded primary care settings in Qatar. BMJ Nutr Prev Health. (2019) 2(1):20. 10.1136/bmjnph-2018-000014 33235953 PMC7678476

[7] Chaabna K Cheema S Abraham A Alrouh H Mamtani R. Adult mortality trends in Qatar, 1989-2015: national population versus migrants. PLoS One. (2018) 13(9):e0203996. 10.1371/journal.pone.0203996 30252887 PMC6155516

[8] Nayak SB Rahming V Raghunanan Y Raghoonath C Rahman A Rajh D Prevalence of diabetes, obesity and dyslipidaemia in persons within high and low income groups living in north and south trinidad. J Clin Diagn Res. (2016) 10(5):IC08. 10.7860/JCDR/2016/18154.7875 27437244 PMC4948420

[9] Obirikorang C Osakunor DNM Anto EO Amponsah SO Adarkwa OK. Obesity and cardio-metabolic risk factors in an urban and rural population in the ashanti region-Ghana: a comparative cross-sectional study. PLoS One. (2015) 10(6):e0129494. 10.1371/journal.pone.0129494 26046349 PMC4457529

[10] Carrère P Fagour C Sportouch D Gane-Troplent F Hélène-Pelage J Lang T Diabetes mellitus and obesity in the French Caribbean: a special vulnerability for women? Women Health. (2018) 58(2):145–59. 10.1080/03630242.2017.1282396 28095137

[11] Kromhout D. Diet and cardiovascular diseases. J Nutr Health Aging. (2001) 5(3):144–9. PMID: 11458283 11458283

[12] Tada H Takamura M Kawashiri M-A. The effect of diet on cardiovascular disease, heart disease, and blood vessels. Nutrients. (2022) 14(2):246. 10.3390/nu14020246 35057427 PMC8780028

[13] de Oliveira Otto MC Alonso A Lee D-H Delclos GL Jenny NS Jiang R Dietary micronutrient intakes are associated with markers of inflammation but not with markers of subclinical atherosclerosis. J Nutr. (2011) 141(8):1508–15. 10.3945/jn.111.138115 21653577 PMC3138642

[14] Maywald M Rink L. Zinc homeostasis and immunosenescence. J Trace Elem Med Biol. (2015) 29:24–30. 10.1016/j.jtemb.2014.06.003 25022332

[15] Ahn B-I Kim MJ Koo HS Seo N Joo N-S Kim Y-S. Serum zinc concentration is inversely associated with insulin resistance but not related with metabolic syndrome in nondiabetic Korean adults. Biol Trace Elem Res. (2014) 160:169–75. 10.1007/s12011-014-0045-1 24943234

[16] Seo J-A Song S-W Han K Lee K-J Kim H-N. The associations between serum zinc levels and metabolic syndrome in the Korean population: findings from the 2010 Korean national health and nutrition examination survey. PloS one. (2014) 9(8):e105990. 10.1371/journal.pone.0105990 25153887 PMC4143320

[17] Medeiros DM. Perspectives on the role and relevance of copper in cardiac disease. Biol Trace Elem Res. (2017) 176:10–9. 10.1007/s12011-016-0807-z 27444302

[18] Klevay LM. Cardiovascular disease from copper deficiency—a history. J Nutr. (2000) 130(2):489S–92S. 10.1093/jn/130.2.489S 10721936

[19] Fukai T Ushio-Fukai M Kaplan JH. Copper transporters and copper chaperones: roles in cardiovascular physiology and disease. American Journal of Physiology-Cell Physiology. (2018) 315(2):C186–201. 10.1152/ajpcell.00132.2018 29874110 PMC6139499

[20] Kang YJ. Copper and homocysteine in cardiovascular diseases. Pharmacol Ther. (2011) 129(3):321–31. 10.1016/j.pharmthera.2010.11.004 21130114

[21] Diez M Cerdà F Arroyo M Balibrea J. Use of the copper/zinc ratio in the diagnosis of lung cancer. Cancer. (1989) 63(4):726–30. 10.1002/1097-0142(19890215)63:4<726::AID-CNCR2820630421>3.0.CO;2-P 2914279

[22] Malavolta M Giacconi R Piacenza F Santarelli L Cipriano C Costarelli L Plasma copper/zinc ratio: an inflammatory/nutritional biomarker as predictor of all-cause mortality in elderly population. Biogerontology. (2010) 11:309–19. 10.1007/s10522-009-9251-1 19821050

[23] Zhang Z Zhao S Wu H Qin W Zhang T Wang Y Cross-sectional study: relationship between serum trace elements and hypertension. J Trace Elem Med Biol. (2022) 69:126893. 10.1016/j.jtemb.2021.126893 34798511

[24] Begum F Me HM Christov M. The role of zinc in cardiovascular disease. Cardiol Rev. (2022) 30(2):100–8. 10.1097/CRD.0000000000000382 35119422

[25] Ozyildirim S Baltaci SB. Cardiovascular diseases and zinc. Biol Trace Elem Res. (2023) 201:1615–26. 10.1007/s12011-022-03292-6 35672544

[26] Xia S Zhang X Zheng S Khanabdali R Kalionis B Wu J An update on inflamm-aging: mechanisms, prevention, and treatment. J Immunol Res. (2016) 2016:8426874. 10.1155/2016/8426874 27493973 PMC4963991

[27] Motamed S Ebrahimi M Safarian M Ghayour-Mobarhan M Mouhebati M Azarpazhouh M Micronutrient intake and the presence of the metabolic syndrome. N Am J Med Sci. (2013) 5(6):377. 10.4103/1947-2714.114171 23923113 PMC3731870

[28] Salgueiro MJ Krebs N Zubillaga MB Weill R Postaire E Lysionek AE Zinc and diabetes mellitus: is there a need of zinc supplementation in diabetes mellitus patients? Biol Trace Elem Res. (2001) 81:215–28. 10.1385/BTER:81:3:215 11575679

[29] Tang X-H Shay NF. Zinc has an insulin-like effect on glucose transport mediated by phosphoinositol-3-kinase and akt in 3T3-L1 fibroblasts and adipocytes. J Nutr. (2001) 131(5):1414–20. 10.1093/jn/131.5.1414 11340092

[30] Little PJ Bhattacharya R Moreyra AE Korichneva IL. Zinc and cardiovascular disease. Nutrition. (2010) 26(11-12):1050–7. 10.1016/j.nut.2010.03.007 20950764

[31] Karagulova G Yue Y Moreyra A Boutjdir M Korichneva I. Protective role of intracellular zinc in myocardial ischemia/reperfusion is associated with preservation of protein kinase C isoforms. J Pharmacol Exp Ther. (2007) 321(2):517–25. 10.1124/jpet.107.119644 17322024

[32] Ford ES. Serum copper concentration and coronary heart disease among US adults. Am J Epidemiol. (2000) 151(12):1182–8. 10.1093/oxfordjournals.aje.a010168 10905530

[33] Urso E Maffia M. Behind the link between copper and angiogenesis: established mechanisms and an overview on the role of vascular copper transport systems. J Vasc Res. (2015) 52(3):172–96. 10.1159/000438485 26484858

[34] Morrell A Tallino S Yu L Burkhead JL. The role of insufficient copper in lipid synthesis and fatty-liver disease. IUBMB Life. (2017) 69(4):263–70. 10.1002/iub.1613 28271632 PMC5619695

[35] Aigner E Weiss G Datz C. Dysregulation of iron and copper homeostasis in nonalcoholic fatty liver. World J Hepatol. (2015) 7(2):177–88. 10.4254/wjh.v7.i2.177 25729473 PMC4342600

[36] Loyke HF. Copper and zinc in experimental hypertension. Biol Trace Elem Res. (1991) 29(1):45–9. 10.1007/BF03032673 1711361

[37] al-Othman AA Rosenstein F Lei KY. Copper deficiency alters plasma pool size, percent composition and concentration of lipoprotein components in rats. J Nutr. (1992) 122(6):1199–204. 10.1093/jn/122.6.1199 1588437

[38] al-Othman AA Rosenstein F Lei KY. Copper deficiency increases in vivo hepatic synthesis of fatty acids, triacylglycerols, and phospholipids in rats. Proc Soc Exp Biol Med. (1993) 204(1):97–103. 10.3181/00379727-204-43640 8372103

[39] Chen X Cai Q Liang R Zhang D Liu X Zhang M Copper homeostasis and copper-induced cell death in the pathogenesis of cardiovascular disease and therapeutic strategies. Cell Death Dis. (2023) 14(2):105. 10.1038/s41419-023-05639-w 36774340 PMC9922317

[40] Tsvetkov P Coy S Petrova B Dreishpoon M Verma A Abdusamad M Copper induces cell death by targeting lipoylated TCA cycle proteins. Science. (2022) 375(6586):1254–61. 10.1126/science.abf0529 35298263 PMC9273333

[41] DiNicolantonio JJ Mangan D O'Keefe JH. Copper deficiency may be a leading cause of ischaemic heart disease. Open Heart. (2018) 5(2):e000784. 10.1136/openhrt-2018-000784 30364437 PMC6196933

[42] Sales MC de Oliveira LP de Araujo Cabral NL de Sousa SES das Gracas Almeida M Lemos T Plasma zinc in institutionalized elderly individuals: relation with immune and cardiometabolic biomarkers. J Trace Elem Med Biol. (2018) 50:615–21. 10.1016/j.jtemb.2018.04.026 29716762

[43] Ho M Baur LA Cowell CT Samman S Garnett SP. Zinc status, dietary zinc intake and metabolic risk in Australian children and adolescents; nepean longitudinal study. Eur J Nutr. (2017) 56(7):2407–14. 10.1007/s00394-016-1280-3 27475431

[44] Knez M Pantovic A Zekovic M Pavlovic Z Glibetic M Zec M. Is there a link between zinc intake and Status with plasma fatty acid profile and desaturase activities in dyslipidemic subjects? Nutrients. (2020) 12(1):93. 10.3390/nu12010093 PMC7019783 31905662

[45] Freitas EP Cunha AT Aquino SL Pedrosa LF Lima SC Lima JG Zinc status biomarkers and cardiometabolic risk factors in metabolic syndrome: a case control study. Nutrients. (2017) 9(2):175. 10.3390/nu9020175 28241426 PMC5331606

[46] Gonoodi K Moslem A Darroudi S Ahmadnezhad M Mazloum Z Tayefi M Serum and dietary zinc and copper in Iranian girls. Clin Biochem. (2018) 54:25–31. 10.1016/j.clinbiochem.2018.02.006 29438682

[47] Song X Wang W Li Z Zhang D. Association between serum copper and serum lipids in adults. Ann Nutr Metab. (2018) 73(4):282–9. 10.1159/000494032 30368500

[48] Darroudi S Saberi-Karimian M Tayefi M Tayefi B Khashyarmanesh Z Fereydouni N Association between hypertension in healthy participants and zinc and copper status: a population-based study. Biol Trace Elem Res. (2019) 190(1):38–44. 10.1007/s12011-018-1518-4 30267309

[49] Bastola MM Locatis C Maisiak R Fontelo P. Selenium, copper, zinc and hypertension: an analysis of the national health and nutrition examination survey (2011-2016). BMC Cardiovasc Disord. (2020) 20(1):45. 10.1186/s12872-020-01355-x 32005161 PMC6995060

[50] Yao J Hu P Zhang D. Associations between copper and zinc and risk of hypertension in US adults. Biol Trace Elem Res. (2018) 186(2):346–53. 10.1007/s12011-018-1320-3 29594690

[51] Malavolta M Piacenza F Basso A Giacconi R Costarelli L Mocchegiani E. Serum copper to zinc ratio: relationship with aging and health status. Mech Ageing Dev. (2015) 151:93–100. 10.1016/j.mad.2015.01.004 25660061

[52] Reunanen A Knekt P Marniemi J Maki J Maatela J Aromaa A. Serum calcium, magnesium, copper and zinc and risk of cardiovascular death. Eur J Clin Nutr. (1996) 50(7):431–7. PMID: 8862478 8862478

[53] Leone N Courbon D Ducimetiere P Zureik M. Zinc, copper, and magnesium and risks for all-cause, cancer, and cardiovascular mortality. Epidemiology. (2006) 17(3):308–14. 10.1097/01.ede.0000209454.41466.b7 16570028

[54] Bill O Mazya MV Michel P Prazeres Moreira T Lambrou D Meyer IA Intima-media thickness and pulsatility index of common carotid arteries in acute ischaemic stroke patients with diabetes mellitus. J Clin Med. (2023) 12(1):246. 10.3390/jcm12010246 PMC9821495 36615047

[55] Shakeri AB Zarrintan S Shakeri-Bavil M. The diagnostic value of the resistivity index of the common carotid arteries in severe internal carotid artery stenosis. Folia Morphol (Warsz. (2008) 67(3):175–8. 10.5603/fm.v67i3.15969 18828098

[56] Stein JH Korcarz CE Hurst RT Lonn E Kendall CB Mohler ER Use of carotid ultrasound to identify subclinical vascular disease and evaluate cardiovascular disease risk: a consensus statement from the American society of echocardiography carotid intima-Media thickness task force. Endorsed by the society for vascular medicine. J Am Soc Echocardiogr. (2008) 21(2):93–111. 10.1016/j.echo.2007.11.011 18261694

[57] Friedewald WT Levy RI Fredrickson DS. Estimation of the concentration of low-density lipoprotein cholesterol in plasma, without use of the preparative ultracentrifuge. Clin Chem. (1972) 18(6):499–502. 10.1093/clinchem/18.6.499 4337382

[58] Unger G Benozzi SF Perruzza F Pennacchiotti GL. Triglycerides and glucose index: a useful indicator of insulin resistance. Endocrinol Nutr. (2014) 61(10):533–40. 10.1016/j.endonu.2014.06.009 25174769

[59] Simental-Mendia LE Rodriguez-Moran M Guerrero-Romero F. The product of fasting glucose and triglycerides as surrogate for identifying insulin resistance in apparently healthy subjects. Metab Syndr Relat Disord. (2008) 6(4):299–304. 10.1089/met.2008.0034 19067533

[60] Bonora E Targher G Alberiche M Bonadonna RC Saggiani F Zenere MB Homeostasis model assessment closely mirrors the glucose clamp technique in the assessment of insulin sensitivity: studies in subjects with various degrees of glucose tolerance and insulin sensitivity. Diabetes Care. (2000) 23(1):57–63. 10.2337/diacare.23.1.57 10857969

[61] Gaziano JM Hennekens CH O'Donnell CJ Breslow JL Buring JE. Fasting triglycerides, high-density lipoprotein, and risk of myocardial infarction. Circulation. (1997) 96(8):2520–5. 10.1161/01.cir.96.8.2520 9355888

[62] Goff DC Jr. Lloyd-Jones DM Bennett G Coady S D'Agostino RB Gibbons R 2013 ACC/AHA guideline on the assessment of cardiovascular risk: a report of the American college of cardiology/American heart association task force on practice guidelines. Circulation. (2014) 129(25 Suppl 2):S49–73. 10.1161/01.cir.0000437741.48606.98 24222018

[63] Stranges S Rayman MP Winther KH Guallar E Cold S Pastor-Barriuso R. Effect of selenium supplementation on changes in HbA1c: results from a multiple-dose, randomized controlled trial. Diabetes Obes Metab. (2019) 21(3):541–9. 10.1111/dom.13549 30280459

[64] Rayman MP Winther KH Pastor-Barriuso R Cold F Thvilum M Stranges S Effect of long-term selenium supplementation on mortality: results from a multiple-dose, randomised controlled trial. Free Radic Biol Med. (2018) 127:46–54. 10.1016/j.freeradbiomed.2018.02.015 29454039

[65] Johnson RW. Alternate forms of the one-way ANOVA F and Kruskal–Wallis test statistics. J Stat Data Sci Educ. (2022) 30(1):82–5. 10.1080/26939169.2021.2025177

[66] Hosmer DW Jr. Lemeshow S Sturdivant RX. Applied logistic regression. Vol. 398. Hoboken, NJ: John Wiley & Sons (2013).

[67] Naik SK Ramanand SJ Ramanand JB. A medley correlation of serum zinc with glycemic parameters in T2DM patients. Indian J Endocrinol Metab. (2019) 23(2):188–92. 10.4103/ijem.IJEM_7_19 31161101 PMC6540900

[68] Bandeira VDS Pires LV Hashimoto LL Alencar LL Almondes KGS Lottenberg SA Association of reduced zinc status with poor glycemic control in individuals with type 2 diabetes mellitus. J Trace Elem Med Biol. (2017) 44:132–6. 10.1016/j.jtemb.2017.07.004 28965568

[69] Ranasinghe P Wathurapatha WS Ishara MH Jayawardana R Galappatthy P Katulanda P Effects of zinc supplementation on serum lipids: a systematic review and meta-analysis. Nutr Metab (Lond. (2015) 12:26. 10.1186/s12986-015-0023-4 26244049 PMC4523910

[70] Jafarnejad S Mahboobi S McFarland LV Taghizadeh M Rahimi F. Meta-analysis: effects of zinc supplementation alone or with multi-nutrients, on glucose control and lipid levels in patients with type 2 diabetes. Prev Nutr Food Sci. (2019) 24(1):8–23. 10.3746/pnf.2019.24.1.8 31008092 PMC6456233

[71] Ruiz-Canela M Hruby A Clish CB Liang L Martinez-Gonzalez MA Hu FB. Comprehensive metabolomic profiling and incident cardiovascular disease: a systematic review. J Am Heart Assoc. (2017) 6(10):e005705. 10.1161/JAHA.117.005705 28963102 PMC5721826

[72] Feskens EJ Virtanen SM Rasanen L Tuomilehto J Stengard J Pekkanen J Dietary factors determining diabetes and impaired glucose tolerance. A 20-year follow-up of the Finnish and Dutch cohorts of the seven countries study. Diabetes Care. (1995) 18(8):1104–12. 10.2337/diacare.18.8.1104 7587845

[73] Mousavi SM Mofrad MD do Nascimento IJB Milajerdi A Mokhtari T Esmaillzadeh A. The effect of zinc supplementation on blood pressure: a systematic review and dose-response meta-analysis of randomized-controlled trials. Eur J Nutr. (2020) 59(5):1815–27. 10.1007/s00394-020-02204-5 32090294

[74] Yang H Liu CN Wolf RM Ralle M Dev S Pierson H Obesity is associated with copper elevation in serum and tissues. Metallomics. (2019) 11(8):1363–71. 10.1039/c9mt00148d 31249997 PMC7753954

[75] He P Li H Liu C Liu M Zhang Z Zhang Y U-shaped association between dietary copper intake and new-onset hypertension. Clin Nutr. (2022) 41(2):536–42. 10.1016/j.clnu.2021.12.037 35030528

[76] Tarantino G Citro V Capone D Gaudiano G Sinatti G Santini SJ Copper concentrations are prevalently associated with antithrombin III, but also with prothrombin time and fibrinogen in patients with liver cirrhosis: a cross-sectional retrospective study. J Trace Elem Med Biol. (2021) 68:126802. 10.1016/j.jtemb.2021.126802 34091123

[77] Jacob R Khan M. Cardiac biomarkers: what is and what can be. Indian J Cardiovasc Dis Women WINCARS. (2018) 3(4):240–4. 10.1055/s-0039-1679104 31934672 PMC6957084

[78] Bayir A Kara H Kiyici A Ozturk B Akyurek F. Levels of selenium, zinc, copper, and cardiac troponin I in serum of patients with acute coronary syndrome. Biol Trace Elem Res. (2013) 154(3):352–6. 10.1007/s12011-013-9754-0 23904327

[79] Emery MP Browning JD O'Dell BL. Impaired hemostasis and platelet function in rats fed low zinc diets based on egg white protein. J Nutr. (1990) 120(9):1062–7. 10.1093/jn/120.9.1062 2398415

[80] O'Dell BL. Role of zinc in plasma membrane function. J Nutr. (2000) 130(5S Suppl):1432S–6S. 10.1093/jn/130.5.1432S 10801956

[81] Vu TT Stafford AR Leslie BA Kim PY Fredenburgh JC Weitz JI. Histidine-rich glycoprotein binds fibrin (ogen) with high affinity and competes with thrombin for binding to the *γ*′-chain. J Biol Chem. (2011) 286(35):30314–23. 10.1074/jbc.M111.253831 21757718 PMC3162390

[82] Tubek S Grzanka P Tubek I. Role of zinc in hemostasis: a review. Biol Trace Elem Res. (2008) 121:1–8. 10.1007/s12011-007-8038-y 17968515

[83] Wang PW Lin TY Hung YC Chang WN Yang PM Chen MH Characterization of fibrinogen as a key modulator in patients with Wilson’s diseases with functional proteomic tools. Int J Mol Sci. (2019) 20(18):4528. 10.3390/ijms20184528 31547461 PMC6770682

[84] Surma S Banach M. Fibrinogen and atherosclerotic cardiovascular diseases-review of the literature and clinical studies. Int J Mol Sci. (2022) 23(1):193. 10.3390/ijms23010193 PMC8745133 35008616

[85] Kunutsor SK Voutilainen A Kurl S Laukkanen JA. Serum copper-to-zinc ratio is associated with heart failure and improves risk prediction in middle-aged and older Caucasian men: a prospective study. Nutr Metab Cardiovasc Dis. (2022) 32(8):1924–35. 10.1016/j.numecd.2022.05.005 35680488

[86] Zhang Wang H Gu S Qiu X Zhang HY. L-shaped association between dietary zinc intake and the risk of developing cardiovascular disease in Chinese adults: a cohort study. Front Nutr. (2023) 10:1032048. 10.3389/fnut.2023.1032048 37006929 PMC10064069

